# Places to Smoke: Exploring Smoking-Related Practices among Danish Adolescents

**DOI:** 10.3390/ijerph18020386

**Published:** 2021-01-06

**Authors:** Stine Glenstrup, Lotus Sofie Bast, Dina Danielsen, Anette Andersen, Tine Tjørnhøj-Thomsen

**Affiliations:** 1 Danish National Institute of Public Health, University of Southern Denmark, Studiestræde 6, 1455 Copenhagen, Denmark; Loni@sdu.dk (L.S.B.); Dida@sdu.dk (D.D.); Titt@sdu.dk (T.T.-T.); 2 Steno Diabetes Center Aarhus, Hedeager 3, 2. sal, 8200 Aarhus, Denmark; Anette.andersen@rm.dk

**Keywords:** school smoking ban, social practice, smoking, youth, smoke-free places

## Abstract

Several established school smoking prevention initiatives involve restrictions on places to smoke. The focus on tobacco control in schools is due to the risk of smoking initiation during adolescence and the perception of this life stage as a period of time when health behavior is established. Hence, this period of time is considered to be ideal for health-promoting initiatives. This paper is part of an ethnographic study on adolescents’ perceptions of tobacco use. Focus groups and field observations were used to explore adolescents’ smoking-related practices related to smoking bans at schools. The findings show that smoking, as a place-based practice, is influenced by locally embedded rules and carries social implications resulting in a distinction between smokers and non-smokers. The distinction between smokers and non-smokers contributes to the retention of a stereotypical view of smokers and, moreover, stigmatizes smokers. According to this, restrictions on places to smoke within the school should be considered carefully in order to avoid stigma or ethical issues.

## 1. Introduction

Despite an overall decline in smoking prevalence in many western countries during the last decades [1], smoking is still the leading cause of premature death and is considered to be one of the leading public health problems [2]. We know that early smoking initiation increases the risk that smoking continues into adulthood; hence, adolescence is considered to be an ideal period of time to prevent smoking initiation [3,4]. Adolescence constitutes a specific period in life when different behaviors are explored and the self is investigated and constructed in the social processes of everyday life [5]. An adolescent’s behavior is nested in a social and historical context and draws from behavior related to both childhood and adulthood. In this respect, behavior (e.g., smoking) often has a distinct social meaning in this specific life stage [5]. Smoking, as well as refraining from smoking, are both considered to be important identity statements [6]. Furthermore, research has found that smoking interferes with other life circumstances, and engaging in, as well as refraining from smoking, can be a way to internalize or resist a stigmatized identity [7]. Smoking identity can be deployed as social power, as smoking is found to help individuals feel included, express membership in their social groups, and maintain an identity that is valued within these groups [8].

Due to the adverse health effects of early smoking initiation, tobacco preventive efforts with youths are considered to be key [9,10,11]. Since attending school is mandatory and it is a place where youth spend a significant number of hours, and the school provides close contact and access to adolescents from different socioeconomic backgrounds and ethnicities, this setting is often chosen as an arena for smoking prevention.

For Danish adolescents, there has been a decline in smoking prevalence since the 1980s, however, this stagnated among school children from 2014 to 2018 [12]. Approximately 5% of 15-year-old adolescents and 3% of 13-year-old adolescents smoke daily, while 17 percent of 15-year-old adolescents and around 5 percent of 13-year-old adolescents smoke at least monthly [12]. As compared with other countries, Denmark has a high smoking prevalence among youth [13]. 

In the World Health Organization (WHO) treaty Framework Convention on Tobacco Control (FCTC), smoke-free policies are recommended as protection from exposure to tobacco [14]. Numerous tobacco control initiatives have been established in school settings [9,15,16,17], several of which comprise smoke-free school policies, either as an independent initiative or in combination with other elements [9,17,18].

It has been suggested that smoking policies at schools have the possibility of decreasing adolescents’ smoking; however, the effects of these policies are inconsistent [15]. For example, a study drawing on data from six European countries found no association between smoking policies at the school and adolescents’ daily smoking [19]. On the contrary, other studies have found that smoking bans, if enforced, decreased smoking prevalence among adolescents [20,21,22]. Various effects of school smoking bans are addressed in a realist review [18]. Here, mechanisms that influenced the effectiveness of the school smoking bans were identified. The results suggested that school smoking bans could reduce pressure to conform to peers’ smoking behavior, and hence reduced the prevalence of smoking. However, a consequence of school smoking bans could be that adolescents find alternative places to smoke, which was suggested to decrease, neutralize, or even reverse the effect of the smoking ban [18]. Another paper reported that school smoking policies decreased smoking on the school grounds but increased the likelihood of students smoking at places other than the school premises [22]. Furthermore, studies have argued that some adolescents approached smoking as a means to rebel against school smoking rules [23,24]. The rebellion against school rules can further divide adolescents into groups of smokers and non-smokers [25]. A recent paper concluded that shared smoking patterns such as reflecting norms and motives for smoking may decrease the effect of school anti-tobacco policies [25]. 

Despite the large number of initiatives comprising smoke-free policies in schools, only a few have actually examined in depth how adolescents perceived and approached such school-based initiatives [25,26]. As in many other studies about youth risk behavior, none of the identified studies incorporated sociological theories [27]. In this paper, we explore how adolescents make sense of such initiatives and, moreover, what possible implications such policies might have by drawing from sociological perspectives. By exploring adolescents’ smoking practices, we seek to understand how smoking-specific places influence smoker/non-smoker dynamics by addressing the meaning of smoking and smoking-specific places. In particular, in this paper, we focus on smoking as a place-based practice and we refer to the social local context of smoking as well as the physical places where smoking is practiced.

## 2. Theoretical Framework

Scholars increasingly argue that more comprehensive knowledge about the social responses to tobacco control policies is vital for understanding smoking behavior and eventually reducing the prevalence of smoking [28,29]. To unfold smoking as a social practice, we apply different theoretical concepts.

We deploy a notion of identity work, as identity work happens through engagement in behavior and actions. Adolescence is defined and constituted in relation to the surrounding and distinct life periods (i.e., child and adulthood). Thus, adolescence is characterized by specific behaviors and ways of acting, all of which influence an adolescence’s self-understanding and sense of identity [5,30]. In [30], Best argued that identity is performed and gives meaning through actions and interactions with others. Thus, identity materializes in youth, in part, by using symbols (e.g., smoking). These symbols can function as boundaries, which define and reinforce social groups [30]. However, as identity (hence, behavior) is given meaning in the interaction with others, the symbolic value of behavior differs according to the specific social context in which adolescents are situated [30].

To explore the influence of social context on the possible differential meaning of smoking among adolescents, we also draw from Frohlich and colleagues’ notion of collective lifestyles [31]. The concept of collective lifestyles was developed to be an expression of the relationship between structures and practices, especially the dialectic relationship between agency (individual action, sense of identity) and structure (rules and resources) [31]. A special focus was how people interacted with rules and resources available [31]. Structure and practices, thus, reproduce behavior and values, i.e., “collective lifestyles are thus an expression of a shared way of relating and acting in a given environment” [31] (p. 791). We used collective lifestyles to investigate the existing local smoking norms, social structure, and agency as determining the social practice of smoking. To fully understand adolescents’ smoking as a place-based practice, we further drew from Setha Low and her theoretical reflection on place and space [32]; and deployed an understanding of place as embodied and socially constructed. The social meaning of place is created by the relationship between the users of the place(s), the circumstances in which they are nested, and the physical construct of the place [32]. Therefore, smoking-specific places are embedded with social meaning, which is produced and reproduced by (non) users. Moreover, the physical form of these places can be interpreted symbolically and transferred to the users of the place [32], meaning that the places where adolescents smoke have symbolic meanings in regard to how smoking is perceived and experienced. We use the concept of translocality to understand the process of moving from one place (e.g., non-smoking place) to another place (e.g., smoking place) as this is perceived to influence identity, creating connectedness and symbolic representation [33]. This provides an analytical perspective on how smoking-specific places contribute to creating different understandings of people, social bonds, and meanings.

## 3. Materials and Methods

The study context for this paper is the Danish school-based intervention “X:IT”. It consists of the following three main components: smoke-free school time (i.e., smoking is not permitted throughout the entire school day), smoke-free curriculum, and parental involvement including smoke-free agreements and chats. The X:IT intervention targets adolescents in grades 7 to 9 (13–15-year-old adolescents). In this paper, we focused on the smoke-free school time. Details about the intervention are published elsewhere [34].

For this paper, a qualitative ethnographic approach was used. The empirical data were comprised of fieldnotes from ethnographic fieldwork carried out in the fall of 2018 and focus group interviews with adolescents as part of the X:IT study II, conducted at the National Institute of Public Health, University of Southern Denmark. The study evaluated the smoking prevention intervention X:IT, designed and implemented by the National Cancer Society, and this paper is a contribution to the evaluation.

### 3.1. The Empirical Context

The empirical data were collected from eighth grade students at two Danish schools. Due to the socially unequal distribution of smoking [35], the schools were selected based on the school’s own assessment of parental educational level. This information was used as a proxy for the socioeconomic position of the adolescents at the two schools. The study focused on eighth graders (14–15-year-old students), as this is the age when many adolescents try to smoke for the first time [12]. 

School A had 373 pupils and one class with grade eight students (N = 20). It was located in a small town, and the students at the school came from nearby towns. Information gathered, during ethnographic fieldwork, revealed that one student was a daily smoker, but smoking was a normal behavior at social gatherings in their leisure time. School B had 460 students and two classes with grade eight students (N = 34). The school was located in a town in which the majority of the children were living. It was observed that none of the students in grade eight smoked, however, other students at the school smoked at places outside the school premises. Although one of the intervention components was aimed at promoting smoke-free school time, both schools practiced smoke-free school premises, which means that students were prohibited from smoking within but not outside the school premises. Thus, smoking during school breaks was allowed. 

Before conducting the fieldwork, parents, teachers, and students were informed about the presence of the researcher, and the parents were given the opportunity to withdraw their children from the study beforehand. 

In Denmark, at the time of the ethnographic study, enforcement of smoke-free school premises was a requirement according to Danish legislation [36].

#### 3.1.1. Participant Observation

Participant observation was chosen as a method to gain information about the adolescents’ (non)smoking practices as well as the social and physical context for their social interactions. The aim was to experience, and thus create an understanding of the world from the view of the students and to create trustworthy relationships [37]. Participant observation took place during classes, breaks, school trips, and some after-school activities. At each school, the students in grade eight were followed over three weeks for 4 to 5 days a week. Field notes were generated for the 28 days of observation and resulted in 120 pages of notes.

#### 3.1.2. Focus Groups

Focus groups were conducted in connection with the observations, since they provided insight into social enactments and the co-creation of meanings and values in context [38]. All grade eight adolescents who were present during the observations were invited to participate in the focus group interviews. Only a few did not want to participate, and a few were not present on the days the interviews were conducted. The number of focus group participants ranged from 4 to 7. In total, 43 adolescents participated in the eight focus groups (four at each school). The composition of the focus groups took into account the social dynamics observed during the fieldwork [38], that is, adolescents who sought each other’s company or partook in similar behaviors such as smoking were assigned to the same focus groups. Due to the linking of smoking and stigmatization [39], and hence the possible sensitivity of the theme [40], prior to the interviews, a guide was conducted, informed by the notion of collective lifestyles, covering themes such as attitudes towards smoking among adolescents and an appreciation of the X:IT intervention. During the focus groups, the adolescents were asked to comment on important events observed during the participant observations. The eight focus groups were transcribed verbatim.

### 3.2. Data Analysis

In this study, we used a combination of data-driven and theory-driven analysis [41]. The interviews and field notes were read thoroughly by the main author several times. Initial open coding involved categorizing and sorting the data into themes using frequently appearing codes. A prominent theme in the material was the replacement of smoking as a reaction to the smoking bans at the schools. The analysis was further informed by the “collective lifestyles” framework, for example, in the development of the interview and observation guide. For instance, the observation guide consisted of questions concerning where, when, and with whom smoking was practiced. This also fostered a focus on places where the adolescents smoked and on the social meaning of these places. Subsequently, the material was reviewed with a focus on differences within and between schools. The analysis of the empirical data was inspired by the concept of collaborative analysis. Hence, to strengthen the analytical findings, the empirical data were discussed with coauthors as well as researchers from different scientific fields and backgrounds [42,43].

## 4. Results

### 4.1. Smoking Practices: (Re)Placing Smoking

Smoking-specific practices taking place outside the school premises existed at both schools. How these distinct places constituted smoking practices is illustrated with School A as a case:

After a few days, I became aware of a behavioral pattern among two students. Every day, Simon and Carlo would leave during break time at 11:15. However, Carlo is absent a lot, and on days when he is not at school, Simon leaves on his own or invites one of the other students from the class to join him. On this particular day, Simon and Kristian leave together. I follow them out of class into the hallway, “Simon and Kristian, may I join you?” Simon looks at me, “Yes, sure you can.” Simon and Kristian start to walk, and I follow them. We walk to the school parking lot. “Where are you going?” I ask. Simon turns to me, “we are heading for the bushes”, he says and gestures with his head towards the end of the parking lot. The parking lot is next to fields, but there is a thicket at the boundary. We go into the bushes so that we can’t be seen from outside. Simon takes a pack of cigarettes from his fanny pack and lights one. Empty cigarette packs are strewn around at the ground, indicating that we are not the first to come here for a smoke. A group of boys from a different grade comes into the bushes. They stand in a short distance from us and all light a cigarette.

(Field note extract, School A)

As the example of the thicket illustrates, smoking was often done discretely, i.e., a hidden-away behavior, practiced at distinct places. The thicket was a place constructed by freely growing bushes and trees out of sight from controlling authorities (i.e., school staff). According to Low [32], the wild and freely growing nature, which constitutes the thicket, can be interpreted as a symbol for the use, as well as the users, of the place. The thicket represents a place where rules (i.e., school smoking ban) do not apply, which becomes congruent with the practice of smoking, as well as smokers, i.e., people acting freely contrary to acting in control. Moreover, as the thicket has a distinct location, it creates a movement for the adolescent smokers from being and sharing the space of the school with all students to sharing and creating a place for smokers with smokers. “It is the ones who smoke who can go there (in the thicket) to smoke but those who do not smoke, cannot join. It is only those who smoke who can go there” (focus group, School A). As a result of this, the thicket was a designated place for smokers to practice smoking. The adolescents act according to and reinforce structures; the smokers by smoking in the thicket, and the non-smokers by refraining from entering.

### 4.2. Smoking Ban: the (Un)Acceptable Smoking Practices

To understand the practice of smoking at distinct and remote places as an embodied space, we examined how this practice reflects the adolescents’ experiences and consciousness. We observed similarities and differences between the adolescents at the schools regarding their practice of smoking and the use of place.

At both schools, the adolescents accepted the schools’ smoking bans, which, in general, were explained by the anticipated influence of smoking in the presence of younger children. This was, among others, explained by a smoker at School A who said, ”You don’t want to show it [smoking] to everyone else, especially minors and people like that, so they think, I’d like to do that too, or something.” Not smoking in the presence of younger children represents a norm concerning smoking and indicates that smoking is an inappropriate behavior for younger children. According to Best [30], smoking represents a symbolic boundary. The practice of smoking is in opposition to being a child, as smoking is perceived as an act to “Feel older... you are just a little bit cooler when you get older” (focus group, School A). Smoking is acknowledged as a marker of age, and hence becomes a practice that carries some opportunities regarding self-representation, positioning, and construction of social identity [30]. Furthermore, when establishing smoking as a behavior delimited to being mature, the act of moving to the thicket represents matureness as opposed to childishness, and as mentioned, being free as opposed to being controlled. Consequently, smoking bans enable agency to be demonstrated by practicing smoking at distinct places, such as in the thicket. This further contributes to recreating the practice of smoking as a behavior for the older. Similarly, the adolescents at School B acknowledge the age-symbolic value of smoking. However, contrary to School A, this meaning of smoking was challenged:

Hellum: I’ve always felt that we were a young class. When we were in the third grade, for example, and looked up to the eighth graders, they just seemed so grown up–smoking and driving scooters. Now we’re up here, and I still feel that we’re young.

Sofie: Not immature but not mature. We’re not like… We don’t drink and all that.

Moderator: Immature?

Sofie: No, not that it’s immature. But those that are older, for example, they finished school some years ago, but they were really forward. I mean, not that we’re immature; we’re mature because we’re careful.

(focus group, School B)

This illustrates the in-between position of adolescence, which seemingly is reinforced by the adolescents’ understanding of the symbols attached to a specific behavior such as smoking and drinking. Moreover, not practicing these behaviors challenges their self-understanding of being young people [30]. As a result, smoking contracts another meaning, i.e., the symbol of refraining from smoking reflects matureness as opposed to smoking which reflects immaturity. The perception that smoking is immature was reflected in the anti-smoking norms at School B, where smoking was not a prominent and normal practice. This was further illustrated in the adolescents’ understandings of and approach to the smoking ban at the school as follows:

Martin: No, not even the cleaning ladies respect it [the smoking rules].

Pelle: No, not at all.

Martin: They are standing right outside the multifield and smoke. They leave the school area, but they [go for a] smoke.

(focus group, School B)

The above excerpt illustrates the existing norms, because even though the cleaning ladies seemingly complied with the formally stated rules, which was not to smoke at the school premises, they were still considered to not comply with the smoking rules. With this, the informal rules of not smoking in visible places (and in front of younger children) represented the smoking norms at School B. Contrarily, the adolescents at School B accepted that smokers could break the smoking ban (i.e., go to the thicket to smoke). This acceptance was further grounded in an understanding of how pervasive the addiction of smoking can be, “People that are addicted have to smoke because they’re addicted, so I think it’s good that they’ve set up a place where they can go and do it” (focus group, School A). The lack of the possibility for smokers to smoke during a school day is considered to have different consequences such as lack of concentration and the possibility of smokers smoking inside the school. This perspective of smoking further expresses sympathy for the smokers and contributes to a perception of smoking (despite a smoking ban) as an acceptable practice when practiced in distinct places. Furthermore, we argue, it reflects local smoking-specific norms, as smoking is a prominent practice that multiple adolescents at School A partake in.

### 4.3. Places to Smoke: The (Anti)Social in Smoking

As the smoking-specific places are remotely located at the schools, the practice of smoking required movement from the school setting to the thicket, which also means that the smokers physically move away from some students but gather with others. Thus, smoking at distinct places has social implications that have the effect of strengthening and hindering social ties:

Simon: We talk to the persons standing over there, and we don’t usually talk to them.

Moderator: Yes?

Simon: That’s how it is, sort of.

Marikka: That’s how you make new friends.

(focus group, School A)

The smoking-specific places had a collective dimension which contributed to socialize the smokers, and thus created specific groupings (i.e., smoking-based friendships), which had some implications for the interactions among peer groups. This group division is characterized by “those who smoke and those who don’t smoke, so both feel a bit like outsiders” (focus group, School A). Smoking, hereby, represented what Best denoted to be a ”symbolic boundary” and contributed to creating a distance between those who participated in smoking and those who did not [30]. This division is expressed by a non-smoking adolescent as, “when we’ve been places, there is a community around those who smoke. They often have something in common with each other that we [non-smokers] can’t be a part of” (focus group, School A). This smoking community not only exists in the context of the school where adolescents, due to structures (formal rules and norms), seek and establish smoking-specific places but transmits to other contexts. The social implications and symbolic boundaries of smoking (and the practice of smoking at distinct places) were further illustrated by Mads, a non-smoker. Mads felt left behind and experienced that the smokers practice smoking during class, as well as during informal breaks between classes.

It’s kind of a shame that your friends just desert you. But I can also perfectly understand that if they’re addicted to smoking now or something, then they should probably be allowed to do it … but it’s a little annoying that they don’t just do it during breaks but in the middle of class. Sometimes we only have a 10-min break before the next lesson starts and then they just go outside and smoke.

(focus group, School A)

This illustrates a friction between the understanding of smoking, on the one hand, as a pervasive addictive behavior (which needs to be practiced) and, on the other hand, as a practice that disrupts the social intercourse between smokers and non-smokers. This disruption, we argue, is a consequence of the distinct places smoking is practiced, and hence smoking adolescents’ movements to these places. The distinct locations of the smoking-specific places and their exclusivity for smokers act as and reinforce symbolic boundaries between peer groups. The boundaries further create a conflict between smokers and non-smokers as the non-smokers feel excluded from the socialization taking part among those who smoke. However, commenting on smoking behavior contributes by exposing and alienating the smokers.

Simon: Yeah, but Mads says it every time, you know. Even if he … I’ve only been gone for two minutes, and he’ll ask, ‘have you just been out smoking?’ That’s a bit strange. Like, a bit…

Marikka: Yeah, he’s like super annoying, so he always has to make some shitty comment.

Moderator: You think it’s annoying that he comments on it?

Carlos: Yes.

Simon: Yes, also just because he talks, even when there are other classes, he talks loudly about it. And it’s not because you don’t want them to know, but that you actually couldn’t care less. But then–why even…?

(focus group, School A)

The fact that the adolescents who smoke express a feeling of discomfort, when their smoking is exposed, indicates that smoking, regardless of being a normal and relatively accepted behavior, has some negative connotations which may challenge the smoker’s self-representation. The replacement of smoking, for instance, the move to the thicket, may function to hide their smoking practice but ends up exposing it. The negative connotation of smokers was illustrated by Ruben who was a non-smoke.

Ruben: It [smoking] might make a worse first impression. No, I don’t know.

Moderator: Yes?

Ruben: it was just to say something. [Laughs]

Fie: Oh Rub [nickname of Ruben]!

Moderator: A bad first impression?

Ruben: Yeah, a little. It probably depends on whether they’re nice when talking to them, then it goes away again quick … It’s just that if there are a lot of people over here smoking, then you might not want to go over to them. Because, yeah, I don’t know.

(focus group, School A)

Despite the general acceptability of smoking at School A, some adolescents still express a perception of smokers as special types of people. This expresses norms and, moreover, results in stigmatizing smokers. The perceived social bond between smokers and the stereotypical view of smokers influence the interactions between non-smokers and smokers. Hereby, practicing smoking becomes a pervasive identity statement. Similarly, the adolescents at School B expressed a tendency to keep a distance from smokers because of their smoking status.

“I feel a bit like… Now I’m just using the ninth grade as an example. If you know that some of the ninth graders smoke, you get a kind of overall idea–a prejudice about what they’re like and, ‘that’s probably not someone you want to talk to because they smoke’.”

(focus group, School B)

Smoking represents, as earlier described, a symbolic boundary. However, in the case of School B, the boundary is influenced by the pervasive anti-smoking norms and a certain perception of the kind of people smokers are.

## 5. Discussion

In this paper, we aimed to explore adolescents’ smoking-related practices in light of smoking bans at schools; particularly, we focused on place(s) to smoke. Our results highlight how an analytical focus on place contributes to an understanding of how the social meaning of smoking, as a place-based practice, is (re)created and further carries social implications. These findings are considered to be valuable, especially considering the focus on smoking prevention among adolescents and the increase in smoking regulation policies in adolescents’ everyday settings.

Our results show that smoking adolescents feel discomfort when their smoking behavior is exposed, indicating that smoking is not well regarded. Thus, when smokers practice smoking at distinct places, it indicates that smoking has a negative connotation. Moreover, many smokers experienc stigmatization [44,45], among others, due to perceptions that smokers have a ”devalued identity” [7] (p. 313). Research has found that adolescents may feel pressured to find alternative places to smoke to demonstrate agency relative to discourses about smoking as an unhealthy behavior [46]. A review by Evans-Polce et al. reported that smokers avoided smoking in public spaces to avoid stigmatization and, further, that smokers who experience stigmatization based on their smoking practice were at risk of being socially isolated [45]. Furthermore, our results suggest that the interactions between smokers and non-smokers is influenced by how smokers are perceived, resulting in some non-smokers being reluctant to interact with smokers, due to their smoking status. This is in line with results from Moore, who found that the negative perception of smokers was ascribed to the entire identity of the person who smokes [47]. Being a smoker can result in loss of status as a consequence of smoking stigmatization [7,39,48]. The adolescents’ smoking practice and the practice of smoking at distinct places might, therefore, be a way to avoid stigmatization. Ironically, smoking at a distinct place that is exclusive for smokers contributes by identifying who smokes and, consequently, cements a smoker’s (and stigmatized) identity.

While some research addresses how smoking is experienced by different and combined social identities such as gender and race, (non)smoking as social identity is not addressed [49,50]. In light of the emphasis of non-smokers on the smokers’ status in this study, our findings underline the importance of this perspective. Thus, smokers can have other social identities with which they experience or have been experiencing disadvantages; smoking can become a behavior to oppress or reform current or former status. Hefler and Carter, for instance, found that for adolescents who had poor academic achievement, smoking had a social aim and was a way to take on another identity, and hence smoking could act as an escape from an already stigmatized identity [7]. In this sense, smoking-specific places, and thus smokers’ limited access to the school ground, as well as smokers’ experience of stigmatization, become an experience that intersects with other circumstances or identities.

As mentioned, our findings further underscore how the practice of smoking at distinct places can create pro-smoking communities, and, conversely, also disrupt the socialization, as the distinct places are exclusively for smokers. This is, among others, supported by Pokhrel et al., who found that young adults initiated using e-cigarettes to join the smokers at the smoking-specific places, but without smoking conventional cigarettes [51]. Although the smokers in our study expressed that the smoking-specific place(s) were places where new friendships arose, the smoking socialization had a downside. Because, as reported in a review by Schreuders et al., the distinct places of smoking can be counterproductive to school smoking bans, since it might contribute to a situation where some adolescents either initiate smoking or continue smoking to preserve a sense of belonging [18]. Being accepted by an immediate social group of smokers but simultaneously stigmatized by society can create fundamental internal conflicts [8]. Others have argued that the social and physical distance between smokers and non-smokers could contribute to non-smokers having less understanding of the lives and experiences of smokers [52,53]. The smokers’ stigmatizing experiences might arise from non-smokers’ feelings of being excluded. We argue this is a consequence of smoking being practiced at distinct places, contributing to alienating smoking and smokers, and hence reinforcing existing stigmatizing perspectives on smokers. Moreover, the ethics and the effect of stigmatization in relation to health promotion has been widely discussed [48,54,55,56,57]. The fact that stigmatization countervails the health promotive initiative is especially a concern. For example, the feeling of stigma decreases the likelihood of successfully quitting smoking [58], which is explained by self-esteem and self-efficacy loss, due to internalizing a smoker’s (hence, stigmatized) identity [45]. Moreover, smoking stigma can possibly isolate smokers, withdrawing them from non-smokers and, consequently, they only socialize with smokers, which could have the effect of reinforcing smoking practices [59]. The stigmatization experienced in the context of the school seems to reflect general attitudes toward smoking and smokers in the wider society.

This study has a few limitations. In general, a low percentage of adolescents smoke. This was also reflected in our sample. The number of adolescents at the two schools who practiced smoking was limited. Thus, the smoker perspective presented in the analysis draws from statements from only a few adolescents. Furthermore, the ethnographic study was undertaken at two secondary schools. The inclusion of more schools could have contributed to more contextual nuances and possibly more adolescents who smoke. Neither of the two schools enrolled in the X:IT intervention had a complete smoking ban during school time. Thus, conducting an analysis of how adolescents interact with a complete smoking ban during school time was not possible, even though this strategy has received political support, among others, in Denmark. Moreover, it is known that different factors such as ethnicity, gender, and socioeconomic status influence socialization [60]. However, the influence of other factors was beyond the scope of this study and the results should be interpreted with this in mind. Nevertheless, as previously mentioned, smoking is a behavior that intersects with characteristics, for example, socioeconomic position [35].

## 6. Conclusions

Overall, the results of the present paper imply that although some studies have shown that banning smoking from the school ground was effective in decreasing smoking, there could be some unintended side effects of such initiatives. These side effects are of an ethical character and, moreover, possibly countervail the effect of a smoking ban. This should be considered when establishing future smoking initiatives. On the basis of the results of the present paper and exciting research, we do not recommend establishing smoking-specific smoking places in school settings. Given the political attention and support to implement smoke-free school time, among others, recently in Denmark, we suggest that future research investigate the possible implications of such an initiative.

## Data Availability

The data are not publicly available due to [participant not providing consent to publicly share data].
